# A Historical Overview of the Role of Benzodiazepines including Clonazepam in the Treatment of Adult Restless Legs Syndrome and Periodic Limb Movements in Sleep

**DOI:** 10.5334/tohm.824

**Published:** 2024-05-02

**Authors:** Arthur S. Walters, Karen Spruyt, Djibril M. Ba, Xiang Gao

**Affiliations:** 1 Vanderbilt University Medical Center, Nashville, TN, USA; 2 UniversitéParis Cité, NeuroDiderot INSERM, France; 3 Penn State College of Medicine, Department of Public Health Sciences, Hershey, PA, USA; 4 School of Public Health, Institute of Nutrition, Fudan University, Shanghai, China

**Keywords:** Restless Legs Syndrome (RLS), Periodic Limb Movements in Sleep (PLMS), benzodiazepines, clonazepam, nitrazepam, temazepam, triazolam, alprazolam

## Abstract

In a recent survey of 16,694 people receiving treatment for Restless Legs Syndrome (RLS), approximately 25% were treated with benzodiazepines either singly or in combination with other RLS treatments. Because of the large number of people receiving benzodiazepines for treatment of RLS, we conducted a historical overview of the therapeutic role of benzodiazepines in RLS and its associated condition Periodic Limb Movements in Sleep (PLMS). We found 17 articles on the use of clonazepam in RLS, PLMS, or both, 3 on triazolam and PLMS, 1 on alprazolam and RLS, 1 on temazepam and PLMS, and 1 on nitrazepam and PLMS. The order of benefit of benzodiazepines from the summarized literature is Sleep>RLS>PLMS and arousals > PLMS. Most of the studies on clonazepam employed dosages of 0.5–2.0 mg. Dosages of 3 or 4 mg caused lethargy, somnolence and confusion. An epidemiological study on the therapy of RLS suggests that treatment of RLS with most types of RLS medications including benzodiazepines in combination with other RLS therapies lowers the future cardiovascular risk associated with RLS. The major effect of benzodiazepines is through potentiation of the effect of GABA on the GABA A receptor. Neuroimaging studies suggest that GABA is altered either positively or negatively in various brain regions in RLS and genetic studies suggest that there are alterations in the GABA receptor in RLS. These results suggest that medications with different GABAergic mechanisms such as tiagabine (Gabitril) or others should be investigated in RLS for their possible therapeutic benefit.

**Highlights:**

Benzodiazepines are frequently used as therapy in Restless Legs Syndrome (RLS) and Periodic Limb Movements in Sleep. The order of benefit is Sleep>RLS>PLMS and arousals > PLMS. For clonazepam dosages of 0.5 mg–2.0 mg/day are most frequently employed. Benzodiazepines exert their therapeutic effect through GABA-ergic mechanisms.

## Introduction

In a recent survey of 16,694 people treated for Restless Legs Syndrome (RLS), 4,037 or almost 25% were receiving benzodiazepines, either singly or in combination [1]. A recent epidemiological study on the therapy of RLS suggests that treatment of RLS with most types of RLS medications including benzodiazepines lowers the future cardiovascular risk associated with RLS—hazard ratio 0.75, (95% CI 0.66–0.84) reduced cardiovascular risk for benzodiazepines used either singly or in combination with other RLS therapies) [1]. A previous meta-analysis of benzodiazepines in RLS found no studies that met the standard for the analysis [2]. However, given the frequent use of benzodiazepines in RLS, a historical overview, as opposed to a meta-analysis, of the use of benzodiazepines in RLS is warranted. To our knowledge, a previous review of this kind has not been done. The current study is meant to close this gap. The current overview summarizes the literature on the use of benzodiazepines for the treatment of RLS as well as the use of benzodiazepines for the treatment of Periodic Limb Movements of Sleep (PLMS) which are a frequent accompaniment of RLS. As clonazepam is so commonly used in RLS, we did not include it in our previous review of the lesser-known treatments for RLS [3].

## Methods

This is a historical overview of the use of benzodiazepines including clonazepam in the treatment of adult RLS and its allied condition, Periodic Limb Movements in Sleep (PLMS). No studies were excluded based on the quality of evidence. In step 1 PubMed and Web of Sciences databases were searched with no time limitation for the following combinations of terms: (a) RLS and also RLS AND benzodiazepines; (b) Periodic Limb Movements of (in) Sleep and also PLMS AND benzodiazepines. Clonazepam, temazepam, triazolam, alprazolam and nitrazepam came up in the search. No other individual benzodiazepines came up on the search. In step 2 an additional search using the names of the individual benzodiazepines was then conducted substituting the name of the individual benzodiazepine for the term “benzodiazepine” in the search. In step 3 additional studies that did not come up on the search were obtained by reviewing the reference lists from those articles that were obtained by the search. Except for articles with ancillary information, we included 17 articles on the use of clonazepam in RLS, PLMS, or both, 3 articles on the use of triazolam in PLMS, 1 article on the use of alprazolam in RLS, 1 article on the use of temazepam in PLMS, and 1 article on the use of nitrazepam in PLMS (Table 1).

**Table 1:** Summary of the use of benzodiazepines in the treatment of RLS/PLMS.


MEDICATION	AUTHOR	YEAR	# SUBJECTS	PSG	TYPE OF STUDY	DOSAGE	RLS	PLMS	PLMS AND AROUSALS	SLEEP	SIDE EFFECTS	ASSOCIATED DISEASES	FAILURE OF OTHER AGENTS

**Clonazepam**													

	Matthews [8]	1979	5 RLS with PLMS	No	OL	1mg before bedtime	↓	↓ subjectively	Not reported	↑ subjectively	Not reported	Not reported	Not reported

	Read and Feest et al [9]	1981	15 RLS	No	OL	0.5 mg at 6 PM and 1/2 hr prior to hs	↓	Not reported	Not reported	Not reported	Lethargy at >3mg in one patient where dosage was increased*	15 with uraemia	temazepam, chlordiazepoxide, diazepam, nitrazepam, and lorazepam

	Oshtory and Vijayan [10]	1980	2 PLMS	Yes	OL	1 mg before bedtime	Not reported	↓ PSG	Not reported	↑ PSG	Not reported	Not reported	barbiturates, diazepam, carbamazepine, tricyclic antidepressants

	Montagna et al [11]	1984	6 RLS with PLMS	Yes	DB crossover	1 mg at 1/2 hr before bedtime	Improvement on 4 point scale	No effect on 4 point scale	Not reported	Improvement on 4 point scale	None	Not reported	iron, flurazepam and lorazepam

	Rousseau and Debatisse [12]	1985	2 PLMS	Yes	OL	Not reported	Not reported	↓ PSG	Not reported	Not reported	Not reported	Not reported	Not reported

	Ohanna et al [13]	1985	20 PLMS	Yes	OL	0.5–2.0 mg at bedtime	Not reported	↓ PSG	No effect	↑ subjectively	Not reported	One patient with RLS	Not reported

	Peled and Lavie [14]	1987	20 PLMS	Yes	DB parallel	0.5–2.0 mg hs	Not reported	↓ PSG	↓ PSG	↑ PSG	One with somnolence and one with dizziness	1 with diabetes mellitus, 2 with mild pyramidal signs	Not reported

	Mitler et al [15]	1986	10 PLMS	Yes	DB crossover	1 mg hs	Not reported	No change in PLMS without arousals on PSG	↓ PSG	↑ PSG and improvement on 7 point scale	Not reported	2 with RLS	Not reported

	Boghen [16]	1980	3 RLS with PLMS	No	OL	0.5 mg 3x day or 0.5 mg hs	↓	↓ subjectively	Not reported	Not reported	None	Not reported	Not reported

	Boghen et al [17]	1986	6 RLS	No	DB cross-over	2 mg in divided dosages	No effect 5 point scale	Not reported	Not reported	Not reported	Somnolence in 3 patients on Clonazepam	Not reported	Not reported

	Montplaisir et al [18]	1985	2 RLS with PLMS	Yes	OL	0.5 mg 1 hour prior to hs	↓	↓ subjectively	Not reported	Not reported	Somnolence and confusion at 4mg in one patient where dosage was increased**	Not reported	Not reported

	Horiguchi et al [19]	1992	15 RLS with PLMS	Yes	OL	0.5–1.5 mg at bedtime	↓	↓ PSG	Not reported	↑ subjectively	Not reported	Not reported	Not reported

	Edinger et al [20]	1996	8 PLMS	Yes	OL	0.5–1.0 mg	Not reported	No effect on PSG	↓ PSG	↑ on sleep Log and insomnia questionnaire	None	Not reported	Not reported

	Saletu et al [21]	2001	26 ; 10 RLS, 16 PLMD	Yes	SB crossover	1 mg	Not reported	No overall effect on PSG	No effect on PSG	↑ PSG and sleep quality scale	Not reported	One with obstructive apnea; one with primary snoring	Not reported

	Manconi [22]	2012	46 RLS with PLMS	Yes	SB parallel	0.5 mg	Improvement on VAS	No effect on PSG	↓ PSG	No effect on most parameters	Mild morning drowsiness in 2 patients	Not reported	Not reported

	Roshi et al [23]	2018	Sample size not given RLS	No	OL	0.5 mg hs	improvement in RLS QoL	Not reported	Not reported	Not reported	Giddiness(44.4%), somnolence(33.3%), constipation(11.1%), gastritis (11.1%)	Not reported	Not reported

	Roshi et al [24]	2019	60 RLS	No	OL	0.5 mg hs	Improvement on IRLS	Not reported	Not reported	Not reported	Giddiness(30%), somnolence(50%), constipation and gastritis (10%)	Not reported	Not reported

**Triazolam**													

	Bonnet and Arand [25]	1990	11 PLMS	Yes	DB crossover	Placebo or 0.125 mg; or 0.25 mg at 30 minutes prior to hs	Not reported	No effect on PSG	No effect on PSG	Improved by MSLT and PSG	Not reported	Not reported	Not reported

	Bonnet and Arand [26]	1991	9 PLMS	Yes	DB crossover	0.125 mg	Not reported	No effect on PSG	No effect on PSG	Improved by MSLT, vigilance test and PSG	None	Not reported	Not reported

	Doghramji et al [27]	1991	15 PLMS	Yes	DB crossover	placebo or 0.25 mg or 0.50 mg	Not reported	No effect on PSG	↓ PSG	Improved by MSLT and PSG	One subject with daytime sedative carry-over	Not reported	Not reported

**Alprazolam**													

	Scharf et al [28]	1986	10 RLS	No	OL	0.5–2.0 mg at bedtime	↓	Not reported	Not reported	Not reported	Not reported	Not reported	Not reported

**Temazepam**													

	Mitler et al [15]	1986	10 PLMS	Yes	DB crossover	30 mg hs	Not reported	No change in PLMS without arousal	↓ PSG	↑ PSG and improvement on 7 point scale	Not reported	2 with RLS	Not reported

**Nitrazepam**													

	Moldofky et al [29]	1986	13 PLMS	Yes	OL	2.5 to 10 mg	Not reported	↓ PSG	↓ PSG	↑ PSG and subjectively	One with sluggishness and impaired concentration	two with hypertension; one with stroke; one with complex partial seizures; one with leg cramps; three with end stage renal disease; two with RLS with migraine or “fibrositis” syndrome; one with iron deficiency anemia and menorrhagia; one with coronary artery disease, chronic low back and lower limb pain	Not reported


See text for detailed description of the double blind studies including age, gender, and duration of the studies. **RLS:** Restless Legs Syndrome; **PLMS:** Periodic Limb Movements in Sleep; **PLMD:** Periodic Limb Movement Disorder; **Arousals:** defined as isolated arousals, arousals associated with PLMS or PLMS associated with arousals – check individual citations for details; **OL:** open label; **DB** = double blind; **SB:** single blind; **hs**. = bedtime; **PSG:** polysomnogram; **CGI-S:** Clinical Global Impression Severity Scale; **VAS:** Visual Analogue Scale; **IRLS:** International Restless Legs Scale; **RLS QoL:** Restless Legs Quality of Life scale; **MSLT:** Multiple Sleep Latency Test * Only one patient had to increase the dosage to as much as 3 mg/day and they experienced extreme somnolence and only partial relief of their symptoms. ** The mother’s RLS symptoms responded to 0.5 mg 1 hour prior to hs but the RLS symptoms and PLMS of the propositus required 4 mg/day.

### Pediatric RLS and PLMS

Several open-label series have documented the potentially beneficial role of benzodiazepines in the treatment of pediatric RLS and PLMS. Because of this and because of the role that benzodiazepines may play in the treatment of adult RLS and PLMS, some authors have recommended the use of benzodiazepines, particularly clonazepam, in children with RLS and PLMS [4,5]. However, to our knowledge, formal studies are lacking. Because of this limitation, we have confined our analysis to adult studies.

## Results

Many of the earlier studies were done before validated instruments for the measurement of RLS severity were available such as the International Restless Legs Scale (IRLS) which was validated in 2003 [6]. However, measurement of PLMS by polysomnography was an established tool by the time the first studies of the use of benzodiazepines on RLS and PLMS were done in the late 70s and early 80s.

### Clonazepam

We found 18 studies that examined the use of clonazepam in RLS and PLMS. Of these 18, one study was excluded as it measured dose equivalencies between pramipexole and clonazepam but did not measure the therapeutic effect of clonazepam compared to baseline [7]. Thus 17 studies were left for analysis [8,9,10,11,12,13,14,15,16,17,18,19,20,21,22,23,24] (Table 1). It should be stated that MATTHEWS WAS THE FIRST to note that a benzodiazepine of any kind, in this case clonazepam, had a potential beneficial effect upon RLS [8]. (Table 1).

#### Rls

Of these 17, ten evaluated the use of clonazepam in RLS and nine reported a benefit of clonazepam on RLS [8,9,11,16,18,19,22,23,24](Table 1). Of these 9 studies showing a benefit of clonazepam on RLS, One was a double-blind crossover polysomnographic study [11], and one was a single blind parallel polysomnographic study [22] (Table 1). The other 7 studies showing benefit were open-label studies. The one negative study reporting no benefit of clonazepam on RLS was a double crossover non-polysomnographic study [17] (Table 1).

#### Plms

Of these 17, thirteen evaluated the use of clonazepam in PLMS and 8 reported benefit [8,10,12,13,14,16,18,19] (Table 1). Of these 8 studies, one was a double-blind parallel polysomnographic study [14]. The other 7 studies reporting benefit were open-label studies, 4 of which provided polysomnographic documentation [10,12,13,19]. Of the 5 negative studies, 2 were double-blind crossover polysomnographic studies [11,15], one was a single-blind crossover polysomnographic study [21], and one was a single blind parallel polysomnographic study [22] (Table 1). The other study showing no benefit was an open-label polysomnographic study [20].

#### PLMS and Arousals

Of these 17 studies, six reported on the use of clonazepam on the association of PLMS and arousals and 4 reported benefit [14,15,20,22]. Of these four, one was a double-blind parallel polysomnographic study [14], one was a double blind crossover polysomnogrphic study [15], and one was a single blind parallel polysomnographic study [22]. The other study reporting benefit was an open-label polysomnographic study [20]. Of the 2 studies reporting no benefit, one was a single blind cross-over polysomnographic study [21] and the other was an open- label polysomnographic study [13] (Table 1).

#### Sleep

Of these 17 studies, ten reported on the use of clonazepam on sleep, and 9 showed improvement [8,10,11,13,14,15,19,20,21]. This is not surprising since one of the major FDA approved indications for benzodiazepines is sleep improvement. Of these 9 studies, two were double blind crossover polysomnographic studies [11,15], one was a double blind parallel polysomnographic study [14], and one was a a single blind crossover polysomnographic study [21](Table 1.). The other 5 studies reporting benefit were open-label studies one of which provided polysomnographic documentation [10]. The one negative study was a single-blind parallel polysomnographic study [22].

### Other Benzodiazepines

Amongst the other benzodiazepines, we found 3 studies on the use of triazolam for the treatment of PLMS, the association of arousals with PLMS and the effect of triazolam on sleep in PLM patients [25,26,27]. None of the subjects from the 3 studies had RLS. All 3 of the studies were double blind crossover polysomnographic studies [25,26,27]. None of the 3 studies showed any improvement in PLMS. One of the studies showed an improvement in the association of arousals with PLMS [27] whereas the other two did not [25,26]. All 3 studies showed an improvement in sleep by PSG and MSLT [25,26,27] (Table 1).

We also found one open-label study showing an improvement in RLS symptoms by alprazolam [28] (Table 1).

Additionally, we found one double-blind cross-over polysomnographic study evaluating the use of temazepam in the treatment of PLMS, the association of PLMS and arousals, and the impact of temazepam on sleep in PLM patients [15]. PLMS were not improved but the PLMS-arousal association and sleep itself were improved [15] (Table 1). Two of the subjects had RLS but the effect of temazepam on RLS was not reported [15].

Furthermore, we found one open-label polysomnographic study examining the impact of nitrazepam on PLMS, the association of arousals with PLMS and the impact of nitrazepam upon sleep. All 3 parameters improved [29] (Table 1). Two of the subjects had RLS but the impact of nitrazepam on RLS was not reported [29].

Details of the individual blinded studies exploring the use of clonazepam and other benzodiazepines in the treatment of RLS, PLMS, PLMS plus arousals, and impact on sleep follow. All of the basic findings from these studies are also summarized in Table 1.

### Clonazepam

In 1984 Montagna et al. performed a double-blind randomized cross-over polysomnographic trial with 3 arms of 1 week duration. Patients had previously failed iron, flurazepam and lorazepam. They treated each of the 6 patients with RLS (ages 44–64, 3 F, 3 M) with clonazepam 1.0 mg, a placebo pill, and leg vibration [11]. Clonazepam showed a more pronounced effect on sleep quality and leg dysesthesias on a 4-point self-rated scale than placebo (p < 0.05). Clonazepam was also statistically significantly more effective than vibration for leg dysesthesias (p < 0.05). The patients also underwent polysomnography and jerking movements of the legs were documented during sleep. The patients were also asked to rate leg jerking on the 4-point scale and no significant effect of treatment was found. Five of the 6 patients preferred clonazepam for the treatment of their symptoms. The article explicitly states that side effects were not present [11].

In 1987 Peled and Lavie did a follow-up double-blind parallel polysomnographic study after the completion of the 1985 open-label study above that they had performed with Ohanna et al. [13,14]. In a parallel group design, they studied the effect of clonazepam on PLMS for one month in 20 subjects with PLMS (12 M ages 30–63 yrs and 8 F age 45–70 yrs). Ten patients were randomized to clonazepam 0.5–2.0 mg hs and 10 randomized to placebo. On polysomnography, clonazepam showed a statistically significant decrease in PLMS/hr sleep (60 before treatment and 34 after treatment p < 0.005) and PLMS arousals/hr sleep (32 before treatment and 13 after treatment p < .017). There was also an improvement in sleep parameters on clonazepam as determined by polysomnography. There was no difference in any of these parameters with placebo. Subjective improvement in insomnia or excessive daytime somnolence also occurred in 7/10 patients on clonazepam. In the clonazepam group, 1 patient complained of increased somnolence, and 1 complained of marked dizziness which led to discontinuation. There was no mention of whether or not any of the patients had accompanying RLS [14].

In 1986 Mitler et al studied 10 subjects (mean age 47.1 years, 4 F, 6 M) with insomnia and Nocturnal myoclonus (PLMS) in a double blind cross-over polysomnographic study and reported that clonazepam 1 mg hs administered for a week improved insomnia on a 7 point scale and objective sleep parameters on polysomnography [15]. The total number of PLMS did not decrease. On the other hand, the PLMS/hr of sleep was statistically significantly decreased with clonazepam (26.1 baseline vs. 17.2 clonazepam p = 0.025). However, on further analysis, clonazepam only suppressed PLMS associated with EEG arousal. Two of the patients also had RLS but there was no mention of whether RLS also improved or not [15]. There was no report as to whether side effects were present or not [15].

In 1986 as a follow-up to their original case reports [16], Boghen et al. conducted a double-blind crossover non-polysomnographic study of 6 RLS patients (age 31–61 yrs, 3 F, 3 M) on gradually increasing dosages of clonazepam up to 2 mg in divided dosages versus placebo after 4 weeks of therapy in each arm [17]. Patients reported the degree of their RLS severity each day on a self-rated scale from 0 (best) to 4 (worst). Three patients improved on clonazepam but 2 of these 3 also improved on placebo. Three patients reported sleepiness as a side effect of clonazepam and 1 reported sleepiness on a placebo. The authors conclude that, despite their earlier positive case reports, clonazepam is not effective in RLS [17]. There was no report as to whether side effects were present or not [17].

In 2001 Saletu et al studied the impact of 1 mg clonazepam on PLMS in 10 RLS patients (ages 34–68; 5 F, 5 M) and 16 patients with PLMD (25–68 years; 9F, 7 M) in a single-blind placebo-controlled cross-over polysomnographic study where each phase lasted 1 day [21]. Compared to the placebo, there was an improvement in sleep efficiency and subjective sleep quality in both the RLS and PLMD groups. Their was a statistically significant decrease in the PLMS index with clonazepam in the PLMD group but no decrease in the PLMS index or PLMS arousal index for the two groups combined. There was no report as to whether the RLS symptoms were improved or not [21]. There was no report as to whether side effects were present or not [21].

In 2012 Manconi administered pramipexole 0.25 mg to one group of 17 RLS patients (13 F, 4 M mean age 55.8 yrs) and clonazepam 0.5 mg to a second group of 15 RLS patients(9F,6M mean age 51.4 yrs) and a placebo to a third group of 14 RLS patients (9F, 5M mean age 53.6 yrs) as a single dose on a single night in a single blind parallel polysomnographic study [22]. Interestingly, there was a dissociation of the PLMS from EEG arousals with both medications, but the effect differed depending on which medication was administered. Pramipexole suppressed PLMS but not arousals whereas clonazepam did the reverse. Based upon the results the authors bring into question the mechanism by means of which PLMS might cause sleep disruption but at the same time suggest dual therapy for the treatment of Periodic Limb Movement Disorder (PLMD). To make matters more complicated, the diagnosis of PLMD requires the presence of clinical sleep disruption which can reasonably be assumed to be due to the PLMS. In many patients, PLMS even with arousals are an epiphenomenon with no associated clinical consequences [30,31]. There was no effect of clonazepam on most sleep parameters in the Manconi et al study and mild morning drowsiness was present as a side effect in 2 patients [22]. RLS was improved on a visual analogue scale [22].

### Other Benzodiazepines

#### Triazolam

We found 3 articles where triazolam had been used in the treatment of PLMS [27,28,29].

In 1990 Bonnet and Arand published their initial 3 armed double-blind cross-over polysomnographic study looking at the effect of triazolam in 11 patients (ages 55–75; 4 F, 7 M) with fragmented sleep due to PLMS and excessive daytime somnolence as documented by MSLT [25]. Patients were assigned randomly to placebo, 0.125 mg triazolam, and 0.25 mg triazolam 30 minutes prior to hs. Patients were on each treatment for 3 nights. The total number of PLMS was unchanged with triazolam as was the number of PLMS associated with arousals but total sleep time and sleep efficiency improved and the total number of stage changes decreased with triazolam. Daytime alertness and performance also improved with triazolam on MSLT and subjective ratings [25]. There was no report as to whether side effects were present or not [25].

In 1991 Bonnet and Arand published a longer-term double blind polysomnographic study of 9 subjects (aged 55–79 years; 2 F, 7 M) who were treated with 0.125 mg of triazolam for 12 weeks [26]. The subjects had the same entry criteria as in their previous study, i.e. patients with fragmented sleep due to PLMS and excessive daytime somnolence as documented by MSLT. The patients were administered a placebo prior to the start of the triazolam treatment and 5 days after the end of the triazolam treatment. Polysomnography was conducted while on triazolam at the 6-week midpoint and 12-week endpoint. The results were similar. The total number of PLMS and the number of PLMS/hr sleep did not change with triazolam nor did PLMS associated with arousals, but daytime performance as measured by a vigilance test and daytime sleepiness as measured by MSLT did improve. Sleep efficiency and total sleep time were also improved on PSG at the 6-week and 12-week time periods. The article explicitly states that there were no adverse reactions or side effects [26].

In 1991 Doghramji et al treated 15 subjects (ages 24–59; 6 F, 9 M) with PLMS and excessive daytime somnolence with triazolam either 0.25 mg or 0.5 mg in a double-blind cross-over polysomnographic study [27]. Patients were treated for 4–7 days with either the regimen above or a placebo and then crossed over to the opposite treatment. A polysomnogram and MSLT were performed on the first and last day of treatment for a total of 4 polysomnograms and 4 MSLTs per patient. None of the patients had RLS. By the last day of treatment, the patients on triazolam had a sleep latency of 9 minutes on MSLT compared to 5.7 minutes on placebo showing that triazolam improved daytime drowsiness. Triazolam also improved sleep architecture and sleep continuity on polysomnography. PLMS/hr sleep did not decrease on triazolam but PLMS-associated arousals/ hr sleep did improve (p = 0.035) [27]. One subject in the study experienced day-time sedative carry-over effect [27].

#### Temazepam

In 1986 in the double blind crossover polysomnographic study mentioned above on the use of clonazepam, Mitler et al. also treated 10 subjects (mean age 47.1 years, 4 F, 6 M) for a week with temazepam 30 mg hs and found results similar to that with clonazepam, i.e. that temazepam improved insomnia and sleep parameters both subjectively and objectively but not the total numbers of PLMS themselves [15]. However, as with clonazepam, the number of PLMS/hr showed a decrease with temazepam (26.1 baseline versus 19.2 temazepam p = 0.05). On the other hand, as with clonazepam, temazepam only decreased the PLMS with EEG arousal and not those without arousal [15]. As aforementioned, 2 of the patients had RLS but no mention was made as to whether temazepam improved RLS or not. There was no mention as to whether side effects were present or not [15].

## Discussion

**Figure 1:**
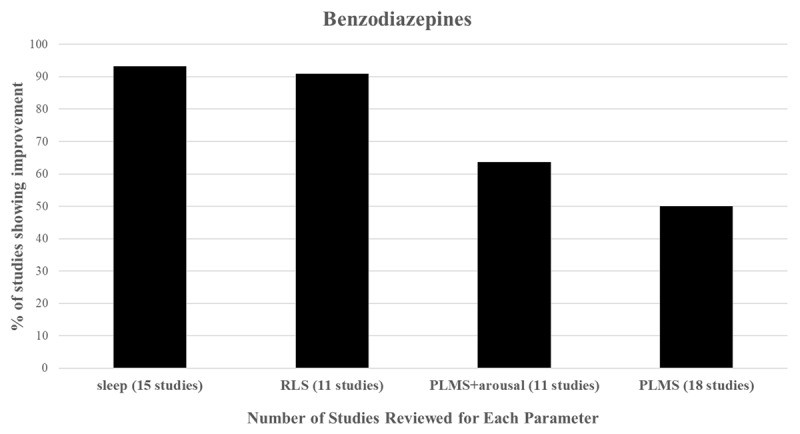
Indicates the number of studies showing benefit for each of the parameters listed for all of the studies of all of the benzodiazepines combined in Table I. An increase in Sleep and a decrease in RLS, PLMS and PLMS associated with arousals are considered benefits. RLS = Restless Legs Syndrome; PLMS = Periodic Limb Movements in Sleep; Arousals = isolated arousals, arousals associated with PLMS or PLMS associated with arousals – check individual citations for details. The order for improvement is Sleep>RLS>PLMS and arousals > PLMS. The order is the same if only blinded studies are taken into account.

We reviewed all published studies on the use of benzodiazepines in adult RLS and PLMS. Table I summarizes all of the studies reviewed. We included 17 articles on the use of clonazepam in RLS, PLMS, or both, 3 articles on the use of triazolam in PLMS, 1 article on the use of alprazolam in RLS, 1 article on the use of temazepam in PLMS, and 1 article on the use of nitrazepam in PLMS. Thus, based on a thorough historical analysis of the literature, it appears that benzodiazepines as a whole may have beneficial impacts (in decreasing order) on sleep (14/15 = 93.3% of studies) > RLS (10/11 studies = 91% of studies) > PLMS and arousals (7/11 = 63.6% of studies) > PLMS(9/18 = 50% of studies) (Figure 1). Taken from the perspective of only looking at blinded studies, the order is the same and the percentages are sleep (8/9 = 88.88% of studies) > RLS (2/3 = 66% of studies)> PLMS and arousals (5/8 = 62.5 % of studies) > PLMS (1/9 = 11% of studies).

For clonazepam, most of the studies employed dosages of 0.5–2.0 mg but dosages of 3 and 4 mg produced lethargy, somnolence and confusion leading to discontinuation (Table 1). At lower dosages for the benzodiazepines reviewed, somnolence, dizziness, confusion anxiety, giddiness, constipation, and gastritis, sluggishness and impaired concentration still occurred in some patients (Table 1). Side effects from the broader literature on the use of benzodiazepines include addiction and tolerance, sedation, irritability, ataxia, and depression [32]. Falls, respiratory failure, an increased risk of car accidents and cognitive consequences have been documented in the elderly on benzodiazepines [33]. However, studies on the long-term use of benzodiazepines in the treatment of RLS and parasomnias, specifically, suggest that tolerance and abuse are low at the smaller dosage ranges if subjects are screened properly [34]. Because of their additive respiratory depressant effect, it is advised the benzodiazepines not be used in combination with opioids.

Clonazepam has been used in epilepsy where the usual maintenance dose is 2–8 mg/day in one or two divided dosages in adults [32]. The initial dose is 0.5–1.0 mg/day titrated upward by 0.5–1.0 mg/week. This raises the possibility that patients were underdosed in some of the studies we reviewed in the literature. On the other hand, given that the patients we reviewed could not tolerate dosages as high as 3 and 4 mg and given that most patients with RLS are older, it may be that the medication is not as well tolerated in this patient population.

In 3 of the studies we reviewed, patients had been unresponsive to other benzodiazepines before clonazepam was successfully used [9,10,11]. These included temazepam [9], chlordiazepoxide [9], diazepam [9,10], nitrazepam [9], lorazepam [9,11], and flurazepam [11] (Table 1). The reasons for this are not clear given the long half-life of clonazepam and the long half life of some of these other agents such as diazepam and flurazepam and given the success of temazepam in PLMS patients reported by other authors [15].

Based upon this summary we make no specific recommendations for the use of benzodiazepines in the treatment of RLS and PLMS but leave it to the individual practitioners to make their own decisions based upon the evidence provided in this article and in the literature as a whole including the previously published meta-analysis [2].

Benzodiazepines act primarily as potentiators of the effect of GABA on GABA A receptors. [35]. The evidence elucidated in this manuscript suggesting that benzodiazepines may improve RLS and PLMS and the fact that this is probably mediated through potentiation of GABA suggests that GABA levels may be low in RLS and PLMS. Neuro-imaging studies suggest that GABA levels are altered in RLS and genetic studies indicate that single nucleotide genetic polymorphisms of the GABA receptor are also altered in RLS [36,37,38]. In an ^1^H-MRS spectroscopy study of 18 RLS patients and a sex-matched control group, brain levels of GABA were not different between patients and controls [36]. On the other hand, GABA levels were positively correlated with PLMS indices and RLS severity in the thalamus but negatively with PLMS indices and RLS severity in the cerebellum. The authors suggest that connections from the cerebellum to the thalamus and then to the striatum are a route of influence [36]. In another MRS spectroscopy study, RLS patients had deficits in cognitive control that were mild, most likely related to working memory deficits [37]. These deficits were less in the RLS patients with higher thalamic GABA levels suggesting that low GABA is pathogenic to some of the cognitive abnormalities that have previously been documented in. RLS [37]. In another study where the most common single nucleotide polymorphisms (SNPs) for the GABA receptor gene were preselected, the frequency of the rs832032T allele of the GABRR3 receptor gene was significantly higher in RLS patients compared to controls [38]. In addition, RLS patients with the GABRA4 rs2229940TT genotype showed a statistically significant younger age of onset of RLS symptoms than those RLS patients with other genotypes [38]. We could not find from the literature that this result had been confirmed in Genome Wide Association Studies (GWAS) of RLS or PLMS. However, in light of these findings, other medications that enhance GABA through varied mechanisms could be entertained as possible therapies for RLS. We became interested in tiagabine (Gabitril) because of its unique mechanism of enhancing GABA levels by inhibition of reuptake of GABA into the presynaptic terminals that released it originally [39]. We have had anecdotal success with the use of tiagabine in the treatment of RLS. Tiagabine was originally designed for treatment of epilepsy but to our knowledge, it has not been studied formally in RLS [40].

Our recent study on the relation of cardiovascular disease to RLS suggests that most of the commonly used medications in RLS including non-ergot dopamine agonists, alpha –2 delta calcium channel acting anticonvulsants such as gabapentin and pregabalin, opioids as well as benzodiazepines could lower future cardiovascular risk in RLS patients [1]. Future studies should further investigate the possible preventative effect of benzodiazepines and other RLS medications on cardiovascular disease.
